# Including environmental and climatic considerations for sustainable coral reef restoration

**DOI:** 10.1371/journal.pbio.3002542

**Published:** 2024-03-19

**Authors:** Heidi L. Burdett, Rebecca Albright, Gavin L. Foster, Tali Mass, Tessa M. Page, Buki Rinkevich, Verena Schoepf, Jacob Silverman, Nicholas A. Kamenos

**Affiliations:** 1 Umeå Marine Sciences Centre, Umeå University, Norrbyn, Sweden; 2 Department of Ecology and Environmental Sciences, Umeå University, Umeå, Sweden; 3 Institute for Biodiversity and Sustainability Science, California Academy of Sciences, San Francisco, California, United States of America; 4 School of Ocean and Earth Science, University of Southampton, National Oceanography Centre Southampton, Southampton, United Kingdom; 5 Department of Marine Biology, The Leon H Charney School of Marine Sciences, University of Haifa, Haifa, Israel; 6 Israel Oceanography and Limnological Research, National Institute of Oceanography, Haifa, Israel; 7 Department of Freshwater and Marine Ecology, Institute for Biodiversity and Ecosystem Dynamics, University of Amsterdam, Amsterdam, the Netherlands; 8 UWA Oceans Institute, University of Western Australia, Perth, Australia

## Abstract

Coral reefs provide ecosystem benefits to millions of people but are threatened by rapid environmental change and ever-increasing human pressures. Restoration is becoming a priority strategy for coral reef conservation, yet implementation remains challenging and it is becoming increasingly apparent that indirect conservation and restoration approaches will not ensure the long-term sustainability of coral reefs. The important role of environmental conditions in restoration practice are currently undervalued, carrying substantial implications for restoration success. Giving paramount importance to environmental conditions, particularly during the pre-restoration planning phase, has the potential to bring about considerable improvements in coral reef restoration and innovation. This Essay argues that restoration risk may be reduced by adopting an environmentally aware perspective that gives historical, contemporary, and future context to restoration decisions. Such an approach will open up new restoration opportunities with improved sustainability that have the capacity to dynamically respond to environmental trajectories.

## Abstract 2

Coral reef restoration faces many challenges. This Essay advocates for prioritizing environmental and climatic considerations to increase the sustainability of future coral reefs and open up opportunities for new restoration approaches.

## Introduction

Coral reefs are one of the most biodiverse ecosystems on Earth, providing goods and services valued at up to $9.9 trillion per year [1]. Nearly 1 billion people live within 100 km of a coral reef [2], a global-scale societal dependence that places significant pressure on coral reef health [3,4]. These acute human pressures are further compounded by anthropogenic climate change, including rapid increases in ocean warming, acidification, deoxygenation, and sea level rise over the coming decades [5]. For corals, migration to more favorable environmental conditions is only possible at the larval stage; settled recruits and established adult colonies must therefore adapt or acclimatize to the environmental conditions they face. However, the capacity for corals to do this is varied [6–8], raising significant concerns about the long-term survival of coral reefs [9] and their capacity to continue delivering ecological and socioeconomic benefits [10].

It is becoming increasingly apparent that indirect conservation and restoration approaches will not ensure the long-term sustainability of coral reefs [11–14], and are “not a viable option for sustainable coral reef management” [15]. Without active interventions, coral reef ecosystems are expected to collapse worldwide within the coming decades [5,16,17], with devastating ecological, socioeconomic, and cultural consequences. Effective reef restoration therefore has the potential to directly contribute to key targets within the UN Sustainable Development Goals, including improved artisanal food security, societal equality, fishery sustainability, and ecosystem resilience [15]. Such potential is exemplified in the recognition of coral reef restoration as a priority action in both the UN Ocean Decade and the UN Restoration Decade initiatives.

Ecological restoration, defined as “the process of assisting the recovery of an ecosystem that has been degraded, damaged, or destroyed” [18], can be a vital practice for habitats where natural recovery is hindered. While the ultimate goal of most restoration initiatives is to return ecosystems to pre-disturbance states, achieving this goal is becoming increasingly challenging in coral reefs [19]. Consequently, efforts to reinstate biological integrity and diversity, ecosystem function, and services may help mitigate long-term impacts. Broadly, restoration activities fall into 2 main categories: indirect restoration (Box 1) approaches that facilitate natural recovery and active restoration (Box 1) approaches that manipulate the system to promote coral growth. However, unless the unfavorable environmental conditions leading to degradation are addressed (which may be feasible for acute stressors such as point-source pollution, but poses a considerable challenge for the pervasive impacts of global climate change), sustainable restoration remains extremely challenging [20]. Further, persistent habitat loss over the long term and the effects of climate change can exceed the adaptive capacities of individual species, leading to fundamental changes in ecosystem structure and/or function [21,22]. Consequently, the pursuit of restoring historical and/or primeval states faces challenges [23–25]. Instead, active restoration initiatives are gaining recognition as essential for safeguarding the future of coral reefs [16,26–28] and their associated goods and services [29], buying time while efforts are made to address global climate challenges.

**Box 1. Glossary**

**Indirect restoration**

Lessen stressors responsible for reef degradation, with the aim of helping natural recovery of coral reefs towards a previous ecosystem state. This approach encompasses actions such as improvements to water quality (e.g., removal of pollution point sources).

**Active restoration**

Deliberate human interventions are taken to expedite reef survival, using specific techniques to manipulate the system and enhance ecosystem function. Examples of such methods include coral gardening and transplantation (sometimes with land-based or ocean-based nurseries), larval enhancement, genetics-based selection, microbiome manipulation, and substratum engineering (i.e., artificial reefs).

**Protection**

Creation of marine protected areas to protect existing reefs from some external pressures (e.g., overfishing). No directed control over the trajectory of coral reef ecosystem change.

**Reef-of-tomorrow**

Novel coral reef ecosystems, established in new areas lacking recent coral reef presence, or in completely deteriorated reefs, featuring distinct communities of coral reef taxa. Reef-of-tomorrow formation is stimulated by implementing active restoration techniques in new and/or degraded areas identified to be within a favorable environmental range for corals (and other associated organisms) in the coming years-to-decades. Reefs-of-tomorrow have the potential to offer a range of ecosystem services comparable to present-day and historical reefs and may create an opportunity for new service provision.

**Environmental envelope**

The set of environments within which it is believed a species or community can persist and their environmental requirements are satisfied.

Nevertheless, despite a clear and pressing need for implementation, coral reef restoration currently faces limitations in widespread scalability, longevity, and cost efficiency [30]. In this Essay, we highlight the undervalued roles of environmental conditions in restoration practice, which carry substantial implications for restoration success. In situ environmental conditions have a well-established correlation with coral abundance and reef development (Table 1), underscoring their crucial importance in “predicting conservation baselines and guiding management investments” [3]. Indeed, a retrospective examination of environmental conditions at reef restoration sites shows that organic carbon, sea surface temperature anomalies, distance from land, and light intensity are some of the most important factors influencing outplant survival [31]. Consequently, giving paramount importance to environmental conditions, particularly during the pre-restoration planning phase, has the potential to bring about considerable improvements in coral reef restoration and innovation. Such environmental and climatic considerations could also be used to inform the most suitable biological and/or (eco)engineering approaches, guiding the selection of novel restoration sites that may not have previously sustained reefs.

**Table 1:** Environmental parameters known to impact some aspects of coral ecophysiology.

Parameter	Coral regulatory capacity	Exemplar references
**Geophysical**		
Temperature	Growth, photosynthesis, respiration, reproduction, recruitment, thermal bleaching, survival, and disease resistance	[8,32–35]
Light intensity (including light pollution at night)	Photosynthesis, calcification, bleaching, chlorophyll content, and symbiont density	[36–39]
Light quality/light spectrum	Growth, coloration, and photophysiology	[40–42]
UV radiation	Cellular and DNA integrity	[43–47]
Lunar light cycles	Reproduction	[45–47]
Waves	Breakage, growth, diffusive boundary layer, and bleaching	[48–51]
Currents	Planulae supply and connectivity	[48–51]
Sedimentation	Bleaching, survival, disease susceptibility, metabolism, calcification, and reproduction	[52–55]
Depth	Refer to light, temperature, carbonate system, nutrients, and waves parameters	[38,56,57]
**Geochemical**		
Salinity	Osmotic stress, photosynthesis, calcification, bleaching, and survival	[58–60]
Nutrients	Calcification, metabolism, bleaching sensitivity, and growth	[61–68]
Pollutants and contaminants (e.g., herbicides, oil)	Photosynthesis and calcification, survival, reproduction, early-life transitions and genetic effects, and metabolism	[65,69–75]
Carbonate system	Calcification, reef growth, bioerosion, bioavailability of other elements, and fish behavior	[66,67,76–82]
Oxygen	Metabolism, bleaching, and survival	[83–87]
Dissolved organic carbon/dissolved organic matter	Microbial biomass, reef microbialization, and dissolved organic carbon release by algae	[88–90]

## Environmental parameter reporting in the coral reef restoration literature

**Fig 1:**
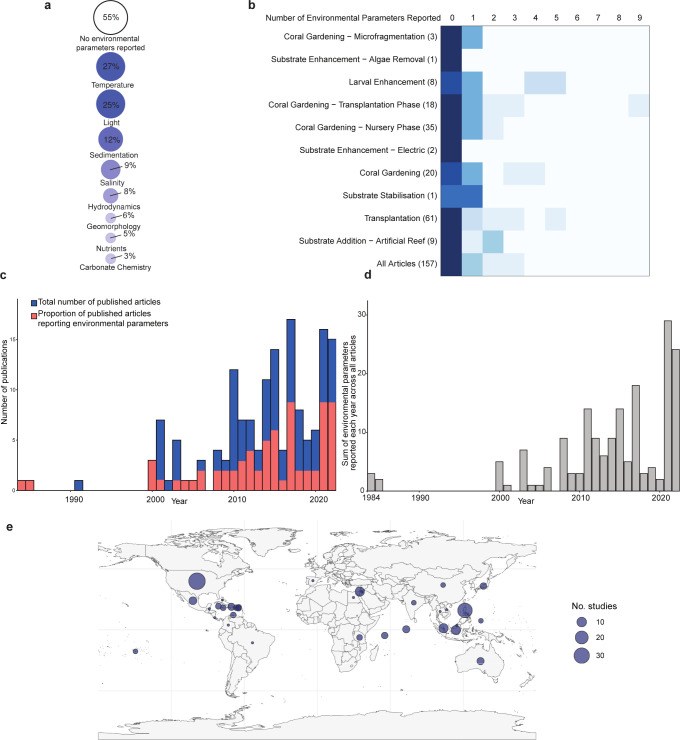
Measurement of environmental parameters in 157 active restoration studies from 1984 to 2022. (**A**) Percentage of articles reporting environmental parameters, grouped according to priority categories suggested by Abrego and colleagues [34,91] (S1 Table, light and depth combined). Depth was included under the “Light” category. (**B**) Heat map indicating the number of environmental parameters reported (columns) in each applied restoration method (rows) in the articles. The total number of articles using a specific restoration method are given after the row title. Color gradient is row specific and proportional to the number of articles corresponding to that restoration method, where the darkest color indicates the highest number of articles within that category. Number of articles for each method are given in parenthesis following the restoration method title, with some articles using more than 1 restoration technique. (**C**) Number of articles reporting measurements of environmental parameters over time. Full height of the bars corresponds to the total number of articles from that year, with the number of articles per year that reported environmental conditions shaded in pink. (**D**) Sum of the number of environmental parameters reported across all articles over time. (**E**) Spatial distribution of articles by location, with points scaled by the number of studies per location. See S1 Table for the literature review search terms and the final publication database.

To provide context on the historical and current prevalence of environmental parameter reporting in coral reef restoration research (before, during, and after restoration activities), we conducted a systematic literature review on academic publications (*n* = 157; S1 Table). Most articles (55%) did not explicitly report any environmental parameters within the manuscript text (S2 Table). Among those that did, temperature and light (provided as depth or light intensity) were the most commonly reported (25% to 27%; Fig 1A and S2 Table); only 30% reported more than 3 parameters (Fig 1B and S2 Table). Despite year-on-year variability, the number of studies reporting environmental parameters and the breadth of parameters assessed over time has increased (Fig 1C and 1D), but with no apparent geographic pattern (Fig 1E). Notably, three critical environmental factors that constrain coral distribution—nutrients, geomorphology, and carbonate chemistry—were each reported in ≤6% of articles (Fig 1A), highlighting a gap in attention of these priority factors [32]. It is important to note that this literature review did not include the gray literature or unpublished results, and it did not consider embedded local knowledge that might not be quantified and documented in primary research manuscripts. Additionally, where environmental parameters were not reported, this does not necessarily mean that environmental parameters were not considered. Nevertheless, our findings underscore the underreporting of environmental parameters in academic literature, which are essential for improving our scientific understanding of the factors that contribute to the success and failure of restoration efforts.

## Environmental and climatic considerations can guide restoration decisions

**Fig 2:**
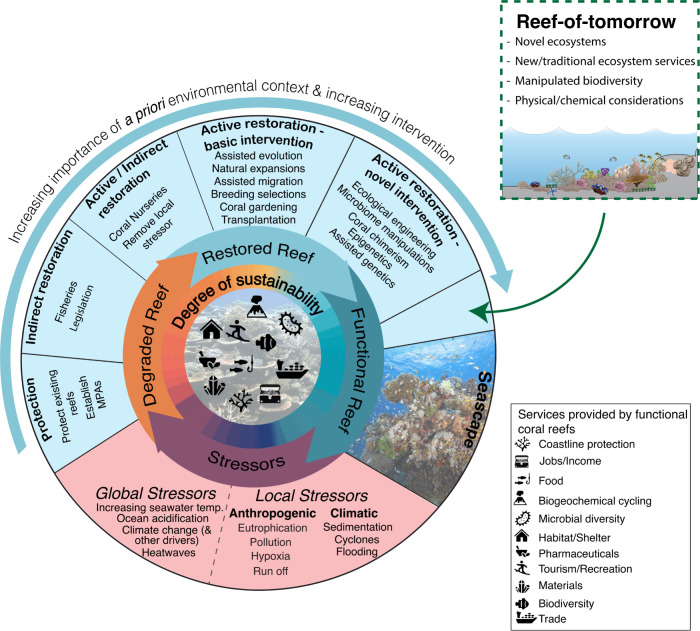
The cycle between functional and degraded reefs, driven by stressors at local–global scales. A return to a functional reef system can be achieved following degradation through a continuum of restoration intervention options (outer band). In the face of a rapidly changing climate, low-intervention options (protection and indirect restoration) are associated with relatively poor long-term sustainability (inner band; red/orange colors) compared to high-intervention options and innovative restoration solutions (inner band; blue/green colors). The gradient of restoration intervention is associated with an increasing necessity for a priori environmental consideration (outer arrow) to guide decisions on the most suitable restoration approach, the best target site(s), and the definition of context-specific restoration success. The reef-of-tomorrow insert proposes a novel restoration intervention strategy, in which future environmental trajectories directly inform the creation of new reef systems that are spatially and ecologically distinct from present-day reefs.

Despite the observed increase in survival rates of restored corals over time [92], environmental factors remain a key determinant of restoration failure, both in the short term and the long term [93,94]. In cases where local environmental changes are minimal, or where human accessibility remains essential for service benefits, restoring coral reefs in current or historic locations with locally derived parent colonies may still be a worthwhile approach, at least in the short term. However, given the widespread impact of environmental change, coastal anthropogenic pressure, and future environmental projections, we propose that environmental considerations should now be given equal weight alongside physiological, ecological, and socioeconomic criteria in restoration decision-making. Adopting a holistic environmental approach enables a departure from the default reactive restoration pattern, allowing for the enhancement of active coral restoration approaches within present-day and projected environmental contexts. This approach creates opportunities for innovative and alternative strategies with higher long-term sustainability (Fig 2).

Achieving the highest level of scientific robustness in coral reef restoration would entail quantifying a comprehensive set of local environmental parameters, complemented by historical context and future projections, which would be applied as priority information layers during decision-making processes. However, the resources, skillsets, logistics, and finances required for such an approach are substantial and likely unattainable in many situations. In light of these constraints, compromises may need to be made, with the understanding that what remains a priority must be locally relevant and guided by contemporary understanding. Prior to applying restoration efforts, the cause(s) of the original reef degradation should be determined so that the most appropriate restoration method can be utilized. To enhance environmental parameter consideration in restoration efforts, we propose three options to aid in navigating the challenges presented.

The first option is to consider the translation of local and traditional environmental knowledge. This is frequently underestimated and tends to go unrecorded in empirical science [20], but it holds considerable potential for addressing gaps in environmental knowledge (particularly concerning spatiotemporal variation). The second option is to access remote data. Satellite remote sensing programs generate extensive datasets across a multitude of parameters. These datasets are particularly pertinent for understanding regional-scale variability, with many programs offering time series spanning several years. To complement this, monitoring initiatives by academic institutions, nongovernmental organizations, and governmental agencies operate in numerous areas. When publicly available or accessible upon request, these datasets provide valuable information for understanding local-scale environmental contexts. In the unlikely circumstance where the first two options are not available, a third option is to conduct baseline environmental monitoring. The selection of parameters to consider depends on the local context (refer to Table 1). Some parameters (e.g., point-source pollution inputs) may require dedicated field efforts for sample collection and analysis. Other parameters are more financially and logistically accessible (Box 2). With this approach, resolving spatiotemporal variability should be a priority to sufficiently inform conservation decisions.

**Box 2. Suggestions for environmental parameter monitoring techniques**

- Secchi disk measurements can serve as a proxy for light conditions and sedimentation.
- Temperature can be easily recorded in situ using low-cost data loggers (less than $40 each).
- Depth (and sometimes temperature) can be easily recorded from dive computers, a common accessory for SCUBA divers.
- Salinity can be measured inexpensively with a hydrometer (under $30) or refractometer (approximately $100). Simultaneous measurement of temperature and salinity enables identification of different water masses, which can be subsequently used to infer water currents and reef oceanography.
- pH (a component of the ocean carbonate system) can be determined at a low-cost with up to 0.1 unit resolution using pH paper strips (around $5 to 10 per 100 strips). In some systems, this may be sufficient to discern spatial and/or temporal variability across a reef. Other components of the carbonate system (e.g., *p*CO_2_, total alkalinity, dissolved inorganic carbon) require greater financial and logistical investment.
- Many of these parameters (e.g., temperature, salinity, pH, turbidity) can also be reliably and potentially continuously monitored using low-cost bespoke sensor systems controlled by micro-computers (for example, Raspberry pi [95,96]).

Once the causes of reef degradation are understood and the environmental seascape across a reef is characterized, decisions regarding the extent and type of restoration intervention must be decided. Historical, contemporary, and future environmental contexts have crucial roles at this stage, gaining significance as the level of intervention increases (Fig 2). In some instances, it may be feasible to eliminate the environmental stressor(s) causing reef degradation (e.g., increased sedimentation from dredging). In these scenarios, adopting an environmentally aware perspective offers robust evidence to support behavioral and/or policy change, and less intrusive options like protection (Box 1) and/or indirect restoration (Fig 2) may prove sufficient to restore a functional reef. In these cases, environmental assessment, conducted through methods such as local knowledge gathering, remote data, and/or baseline monitoring may be confined to a defined period pre-restoration, and focus on a limited suite of parameters related to the specific stressor(s).

## Using environmental considerations to innovate restoration approaches

In cases where the environmental context does not favor indirect restoration approaches, active restoration interventions are required. Although these interventions are inherently more complex and pose greater risks during implementation, their prevalence in the literature is increasing (S1 Table). This may be attributed to the urgent need for climate action, with a rising awareness of the rapidly diminishing and unfavorable projections for the future survival of coral reefs [16,97]. As the extent of restoration intervention expands, both contemporary and future environmental conditions gain heightened significance (Fig 2) and should thus be prioritized during the planning stage. A comprehensive environmentally aware perspective will facilitate a quantitatively informed selection of the restoration location and/or intervention type. This choice can be iteratively refined over time as new data and technologies emerge, which will help to overcome present-day uncertainty in environmental prediction modeling, which remains a fundamental challenge. Nevertheless, an environmentally informed analysis, even without future predictions, would enhance the efficiency of restoration activity resources and contribute to improved sustainability. This approach might include:

1. The identification of restoration areas where an environmental stress is (or will be) lower.
2. The identification of restoration areas where environmental variability is naturally high, providing resilience against background environmental change.
3. The selection of an intervention type that aligns best with the in situ conditions of the restoration area.
4. The selection of coral species that are best suited to the restoration area and to the intervention type.

One potential outcome of such an approach is that restoration could successfully be performed in areas where coral reefs have not been historically present. We propose that these so-called “reefs-of-tomorrow” (Box 1) could prompt a shift in restoration decision-making to something more adaptive to prevailing and projected environmental conditions. If designed appropriately, these reefs-of-tomorrow would ideally provide the same ecosystem services as current reefs, if not more, with enhanced tolerance to current and projected environmental conditions. Active interventions in reefs-of-tomorrow could include both spatial and ecological applications.

Spatially, climate change is causing areas previously suitable for coral growth to become compromised, while regions that were once unsuitable are now, or soon will be, within environmental envelopes (Box 1) conducive to coral reef development [98]. Consequently, there is potential for a latitudinal expansion of coral reefs, particularly in current marginal reef systems; in fact, some coral species are already undergoing poleward migration [99]. This shift opens up ecological, socioeconomic, and cultural opportunities for sustainable coral reef systems over the coming decades, albeit geographically shifted away from current/historical boundaries of coral reef extent. Drawing insights from terrestrial forestry, embedment of environmental considerations into restoration and forest expansion initiatives has improved restoration effectiveness in the former forest margins, which are now within optimal environmental envelopes [100]. Similar principles could be applied in coral reef restoration. To do this, we must recognize that although a reef used to be in a particular location, it might now, or in the near future, be more effective to restore the reef at or beyond its current margins, taking advantage of shifted boundaries in favorable environmental and climatic envelopes. It is important to note that poleward species and habitat shifts are expected to be variable and not a simple increase in latitude [98,101,102]. Rigorous spatiotemporal environmental understanding is essential to determine new envelope boundaries. As uncertainty in environmental projections improves, contingent on the quality of environmental input data, our confidence in predicting future coral (reef) boundaries will also increase. These projection data in turn provide the foundation for guiding the boundaries of optimal restoration sites.

Ecologically, coral reef species exhibit differential responses to the same environmental perturbation, with varying effects on physiological processes including calcification, growth rate, metabolism, feeding behavior, and reproduction, among others (Table 1). Active restoration initiatives typically use either asexual methods (coral transplantation) or sexual recruits, obtained through larval collection and settlement. Among these reef restoration approaches, branching coral species have emerged as the preferred candidates due to their fast growth rates, higher nursery survival rates, the ease of handling large numbers of fragments in the nurseries, esthetic appeal, and the capacity to quickly create complex environments. Considering future environmental contexts, these reef restoration approaches may be further optimized by incorporating naturally heat-resistant corals [103,104], or those engineered to be more environmentally resilient via selective breeding, coral chimerism, epigenetics, and/or microbiome manipulation [105,106], or by artificially increasing structural complexity [107]. This optimization can be employed to design reef-of-tomorrow restoration in a degraded coral reef area, featuring a different consortia of coral reef taxa compared to the pre-degradation state.

Given the rapid rate of global environmental change and the bleak outlook for present-day coral reef distributions, the present moment calls for an expansion in the scope of reef restoration intervention [108], along with a serious contemplation of innovative reef-of-tomorrow strategies (Fig 2). From the standpoint of coral survival, this approach might be the pinnacle in restoration intervention, involving the establishment of new reef ecosystems that are spatially and ecologically distinct from present-day reefs. These would be situated in areas where emerging environmental envelopes are, or soon will be, conductive to sustainable coral reef development. Naturally, these shifts would necessitate consideration of concurrent changes in associated flora and fauna, as well as the implications for ecosystem service provision. The shift towards anthropogenically created reefs, encompassing changes in both ecological composition and location, builds upon the momentum gained from the use of “designer” ecosystems as a tool for goal-orientated, forward-looking restoration practices [109]. While this approach has stirred division within conservation science, proponents argue that it might be “the only way to practically confront novel environmental regimes” [110]. The reef-of-tomorrow restoration approach provides a platform for reef sites with environmentally extreme conditions (e.g., tide pools, back reefs, CO_2_ vent sites, lagoons, mangrove forests, high latitudes) to emerge as crucial areas for the long-term sustainability of the global coral reef ecosystem [111]. Although we recognize these ecosystems, and the corals supporting them, as natural resilience hotspots [104,112], their use as a stress-tolerant coral stock has yet to be fully integrated into active restoration efforts [104] or global conservation initiatives [113]. Nevertheless, small-scale studies are starting to provide evidence that a reef-of-tomorrow approach may be a viable restoration strategy. For example, “bleaching-resistant” coral nurseries have been established 2 km from their tide-pool parent colonies in American Samoa [114], and macrotidal heat-resistant corals from northwest Australia are known to maintain heat tolerance alongside low temperature acclimation [115].

## Using environmental and climatic considerations in a risk–benefit analysis

**Fig 3:**
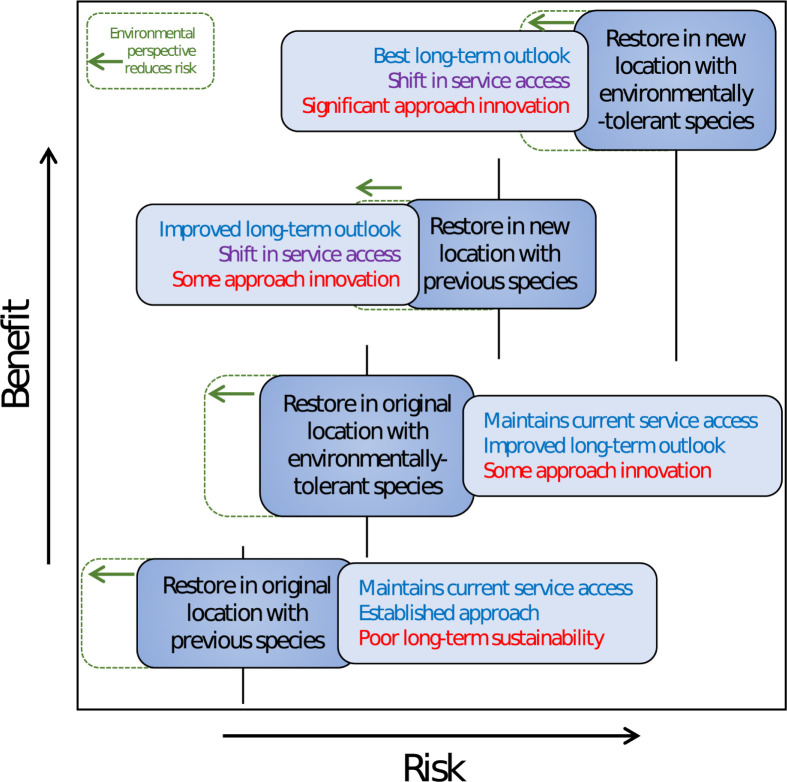
Conceptual overview of a coral restoration risk–benefit gradient. Different restoration approaches (dark blue boxes) have different levels of risk associated with their implementation and long-term sustainability, but also yield differing levels of service benefits (light blue boxes), with implications that are positive (blue text), negative (red text), or mixed (purple text). Shifts in service access may be detrimental to original stakeholders but provide opportunities for new stakeholders. An environmentally aware perspective has the potential to reduce risks while maintaining the benefits (green dashed boxes).

Attempting to predict and select species survivors instead of conserving biological diversity from which survivors could naturally emerge presents inherent risks such as fitness trade-offs and pathogen spread, and may underestimate the adaptive potential of reef communities [109,116]. However, in the face of rapid climate change, the argument for adopting riskier restoration interventions is gaining traction [97,104]. To mitigate these risks, we put forward the notion that in a reef-of-tomorrow approach, the selection of restoration sites and methodologies should heavily rely on a robust, quantifiable environmental foundation. This foundation should encompass a comprehensive understanding of past and present-day environmental conditions, along with high-confidence, low-uncertainty projections of future environmental trajectories, preferably at regional-to-local scales (Fig 3). Environmental contexts will also have a crucial role in guiding and refining restoration goals, as different restoration outcomes will be associated with different environmental optima. The ultimate restoration initiative will represent the delicate balance between service provisions, maximal sustainability, and the priorities of restoration goals. High-intervention restoration approaches that establish new spatial boundaries and/or introduce new ecological communities could yield significant ecological, social, economic, cultural, political, and ethical implications [110], whether positive or negative. The incorporation of these factors in the restoration objectives might lead to the adoption of an environmentally suboptimal approach [100]. However, we emphasize that if this is the case, it should be grounded in a robust environmental knowledge base to minimize inherent risks. Therefore, the success of reef-of-tomorrow restoration relies on a thorough analysis of the risks and benefits associated with both natural and social factors, including environmental trajectories, biodiversity, community accessibility, and service provision. To effectively manage this, restoration objective(s) must be well defined and justifiable across stakeholders [117]. Consequently, stakeholder consultation and consensus building in the risk–benefit analysis remain essential components of restoration planning.

The optimization of any of the restoration approaches presented in Fig 2 faces challenges due to the predictive risk and uncertainty associated with all environmental parameters, particularly when restoration objectives are not well defined. This challenge is especially pronounced for extreme events or unexpected environmental trajectories: the influence of catastrophic events such as marine heatwaves and tropical storms on restoration success for coral reefs and beyond is well documented [118]. Directly mitigating against these events, particularly at a local scale, may not be feasible. To minimize the risk of failure, especially against extreme events, a sensible strategy is to adopt a bet-hedging “portfolio” approach to restoration. This approach involves integrating biological resilience and innovative techniques with environmental considerations to encompass a significant portion of the risk–benefit gradient (Fig 3) and maximize the opportunities for success [109]. Such an approach may involve using multiple species, life stages, and genotypes; implementing restoration at multiple sites or habitat types; and the use of several parallel restoration methods [118,119]. An environmentally aware, bet-hedging reef restoration could therefore operate as a “synergistic intervention” [120], with ethical, political, economic, and ecological justification, and the potential to achieve both social and ecological resilience in parallel.

## Conclusion

Coral reefs stand out as one of the most ecologically, environmentally, and socioeconomically important ecosystems in the marine realm, offering a myriad of direct ecosystem services and provisions to millions of people worldwide. However, rapid environmental changes and escalating human pressures on coastal zones are threatening the enduring survival of coral reefs. It has become evident that relying solely on climate mitigation may not be sufficient to safeguard the future viability of corals and coral reefs. Simultaneously, targeted active restoration actions are deemed necessary, but the long-term sustainability of current efforts raises uncertainties. Environmental factors consistently hold a pivotal role in determining restoration success, yet they are underreported in the existing reef restoration literature. Integrating environmental considerations into restoration strategies will reduce risk and accelerate innovation, as seen in the development of reefs-of-tomorrow, which work with environmental changes rather than working against them. Although not without challenges, we contend that embracing an environmentally aware perspective has the potential to enhance the long-term sustainability of corals and coral reef ecosystems.

## Supporting information

S1 Table Results from literature review of articles from 1984–2022. Research articles from 1984–2022 were identified from Web of Science using the search string “coral* + restoration” within the title, keywords, and/or abstract of publications, and subsequently filtered only for experimental studies involving coral restoration. The environmental parameters that were noted in each article’s methodology as being explicitly measured were recorded. Although water depth of study sites was noted in many articles, this was only counted when explicitly considered as a distinct research parameter (i.e., where comparisons in depth were experimentally investigated).
(XLSX)

S2 Table Measured environmental (i.e., light, depth, temperature, nutrients, pH) variables considered in the 157 reviewed articles were placed into categories defined by Abrego and colleagues. Categories are adapted from the 6 chemical and physical factors that limit the distribution of corals (Abrego and colleagues) and 2 additional categories (i.e., Salinity and Geomorphology). Black boxes show if a factor/category was considered in the reviewed study, and 42 studies considered temperature, 40 light, 19 sediments, 14 salinity, 12 hydrodynamics, 9 geomorphology, 8 nutrients, and 5 carbonate chemistry. ID of the article is listed and can be linked to the specific publication in S1 Table. Carbonate chemistry includes measurements of pH, alkalinity, aragonite saturation, *p*CO_2_, and/or dissolved inorganic carbon.
(XLSX)
